# Prevalence of Foot Diseases and Injuries and Their Associations with Demographic and Health-Related Factors Among Umrah Pilgrims in 2024 G (1445 H)

**DOI:** 10.3390/ijerph22091402

**Published:** 2025-09-08

**Authors:** Ghadah Sulaiman Alsaleh, Bayan Hashim Alsharif, Fahad A. Alamri, Jumanah Alhazmi, Lamis Alabdullatif, Anas Khan

**Affiliations:** 1 The Global Centre for Mass Gatherings Medicine, Ministry of Health, Riyadh 12382, Saudi Arabiakhanaa@moh.gov.sa (A.K.); 2 Hajj and Umrah Research and Epidemiology Administration, King Abdullah Medical, Makkah 24246, Saudi Arabia; alsharif.b@kamc.med.sa; 3 Family Medicine, Primary Health Centre, the Global Centre for Mass Gatherings Medicine, Ministry of Health, Riyadh 12382, Saudi Arabia; faabalamri@moh.gov.sa; 4 Department of Emergency Medicine, College of Medicine, King Saud University, Riyadh 24331, Saudi Arabia

**Keywords:** Umrah, foot injuries, foot injuries, chronic diseases, preventive measures, pilgrim

## Abstract

**Background**: Foot injuries are common among Umrah pilgrims due to prolonged walking, overcrowded conditions, and inadequate preventive measures, such as inappropriate footwear or walking barefoot. Despite their potential impact on mobility and overall pilgrimage experience, these conditions remain underreported and insufficiently addressed in public health strategies. **Objectives**: This study aims to assess the prevalence and types of foot problems among Umrah pilgrims, examine their associations with demographic characteristics and comorbidities, analyze the utilization of medical attention for foot pain, and assess the use of preventive measures to reduce foot-related health risks during the pilgrimage. **Methods**: A cross-sectional study was conducted throughout the 2024 G (1445 H) Umrah season at the Grand Mosque, Makkah. The study recruited 1138 Umrah pilgrims aged 18 and older who performed the pilgrimage. A structured questionnaire was administered to collect data on demographic characteristics, chronic diseases, foot conditions, medical-attention-seeking behavior, and preventive practices. Pilgrims with pre-existing foot conditions were excluded from participation. **Results**: Foot diseases were reported by 46% of participants. The most common foot injuries included sprains/strains (18.7%) and muscle pain/cramps (4.9%), with the leg and forefoot being the most affected areas. Significant associations were observed between foot diseases and lower education levels (*p* = 0.03), chronic liver disease (*p* = 0.04), and cardiovascular disease (*p* = 0.04). Despite the high prevalence of foot-related conditions, only 9.6% sought medical attention, and 14.9% reported using preventive measures. **Conclusions**: The study highlights a substantial burden of foot problems among Umrah pilgrims, with limited utilization of healthcare services and preventive strategies. Targeted interventions, including educational campaigns and improved screening for high-risk individuals, are essential for enhancing foot health and ensuring a safer pilgrimage experience.

## 1. Introduction

The Umrah pilgrimage is among millions of Muslims’ annual religious journeys. Unlike Hajj, which follows a specific Islamic calendar schedule and occurs once annually, Umrah can be performed at any time, leading to a continuous influx of pilgrims throughout the year [1]. Umrah involves several key rituals centered in Makkah, including Tawaf (circumambulation of the Kaaba) and Sa’i (walking between Safa and Marwah hills). Pilgrims typically walk approximately 4–5 km during these rituals, with distances varying based on crowd density and individual paths taken [2,3]. The Kingdom of Saudi Arabia has reported a steady increase in Umrah pilgrims, with over 10 million arrivals in 2023. This number is expected to rise with increasing travel accessibility and governmental initiatives to expand religious tourism under Saudi Arabia’s Vision 2030 initiative [4]. While Umrah’s spiritual and cultural significance is widely acknowledged, its public health implications remain an important area of research, particularly concerning the physical strain experienced by pilgrims [1,5].

Foot diseases and injuries, encompassing both acute conditions (e.g., sprains, fractures, blisters) and chronic pathologies (e.g., ulcers, infections, deformities), are among the most frequently reported medical complaints during pilgrimages. The rituals of Umrah require extensive walking, on hard surfaces, in extreme temperatures and highly crowded environments. Many pilgrims wear inappropriate or ill-fitting footwear, exacerbating their risk of musculoskeletal pain, foot ulcers, blisters, and infections [6,7]. These conditions directly contribute to foot damage through several mechanisms: prolonged walking on hard surfaces transmits repetitive force, leading to stress injuries and plantar pain; ill-fitting footwear creates friction points, causing blisters and ulcers, especially over bony prominences; and extreme heat and humidity macerate skin, increasing susceptibility to fungal and bacterial infections [8,9,10,11]. Diabetic pilgrims face particularly high risks of foot injuries during the pilgrimage. Some discontinue their medications for various reasons, including misconceptions about temporary discontinuation being safe while they are in Makkah, excessive focus on spiritual activities leading to neglected self-care, or practical challenges in accessing medications during rituals. These medication lapses can significantly worsen pre-existing foot conditions [12,13,14,15,16]. This highlights the burden of foot diseases among pilgrims; however, most available data focus on Hajj, leaving a significant gap in knowledge regarding Umrah-specific risks and health outcomes.

The distinct characteristics of Umrah necessitate dedicated research into its specific health challenges. Unlike Hajj, which is concentrated within a particular timeframe and allows for more structured health surveillance [17], Umrah is performed annually by a more diverse demographic of pilgrims, including elderly individuals and those with pre-existing health conditions [18]. The lack of seasonal constraints means that environmental factors, such as extreme heat in summer months or increased congestion during Ramadan, may further influence the prevalence and severity of foot diseases and injuries [19,20]. While healthcare services in Saudi Arabia are well-equipped to support pilgrims, the continuous nature of Umrah presents unique challenges in monitoring and addressing pilgrims’ health needs in real time. Unlike Hajj, where large-scale health initiatives are implemented during a defined period, Umrah pilgrims arrive throughout the year, requiring adaptable healthcare strategies to ensure timely medical attention. Moreover, the variability in pilgrimage density throughout the year, with peaks during religiously significant months such as Ramadan, may impact the strain on available medical resources and infrastructure [21,22]. Pilgrims with chronic conditions, such as diabetes and vascular diseases, may require closer medical monitoring to prevent complications from prolonged walking and inadequate foot care [13,14,15]. Enhancing awareness of preventive strategies, such as proper footwear selection and foot hygiene, is essential for reducing the burden of foot-related ailments. Moreover, understanding the specific health risks associated with Umrah is crucial for optimizing preventive measures and healthcare services to support the well-being of pilgrims [20].

Addressing this knowledge gap is essential for developing effective prevention and intervention strategies. This study aims to assess the prevalence and types of foot problems among Umrah pilgrims, examine their associations with demographic characteristics and comorbidities, analyze the utilization of medical attention for foot pain, and assess the use of preventive measures to reduce foot-related health risks during the pilgrimage.

## 2. Methods and Materials

### 2.1. Study Design and Participants

This cross-sectional study was conducted throughout 2024 G (1445 H), focusing on Umrah pilgrims aged 18 years or older who were actively engaged in the pilgrimage at the Grand Mosque in Makkah, the primary site for performing Umrah rituals. The Grand Mosque, one of Islam’s most significant and holiest mosques, is located in the heart of Makkah and serves as the focal point for Umrah.

### 2.2. Sample Size

The required sample size for this study was calculated using the Raosoft^®^ sample size calculator (Maple Tech, The Woodlands, TX, USA). Assuming a 95% confidence level, a 50% expected response rate, and a 5% margin of error, the initial calculated minimum sample size was 385 participants. Ultimately, the study recruited 1138 participants, significantly exceeding the target sample size.

### 2.3. Inclusion and Exclusion Criteria

The study included Umrah pilgrims aged 18 years or older willing to participate in the research and provide informed consent. Participants had to be actively engaged in the 2024 G (1445 H) Umrah pilgrimage and physically capable of performing pilgrimage rituals. Only individuals who could understand the survey questions and respond in Arabic or English were included. Pilgrims with any foot-related medical condition before the pilgrimage, such as severe diabetic foot ulcers, active infections, or recent surgeries, were excluded from the study. Individuals who could not participate due to cognitive impairments or those who declined participation were also excluded. This ensured that the study sample represented a general population of Umrah pilgrims without significant pre-existing foot-related health issues.

### 2.4. Procedure and Data Collection

Data for this study were collected through a structured bilingual (English/Arabic) questionnaire administered to Umrah pilgrims at the Grand Mosque, Makkah, throughout 2024 G (1445 H) Umrah season. Trained research assistants implemented an assisted self-reporting approach, providing standardized explanations of medical terms when needed. The questionnaire consisted of several sections to assess key variables relevant to the study. The first section gathered demographic characteristics, including information about age, gender, body mass index (BMI), nationality, country of residence, education level, employment status, and prior experience with Umrah or Hajj. The second section focused on the prevalence of chronic diseases, such as endocrine disorders, cardiovascular disease, lung disease, and other comorbidities. The third section addressed the prevalence of foot problems, identifying conditions like sprains, muscle pain, blisters, and fractures, along with their location on the foot. The fourth section explored the utilization of medical attention for foot pain and preventive measures to avoid foot injuries, including practices like using analgesics, special footwear, and wheelchairs.

### 2.5. Ethical Considerations

This study was conducted following the ethical principles outlined in the Declaration of Helsinki, which ensure the protection of the rights, safety, and well-being of participants involved in clinical research. Ethical approval was granted by the Research Ethics Committee at the Ministry of Health, Kingdom Saudi Arabia, with the approval documented under IRB log Number: 24-16 M. Participation in this study was voluntary, and all participants provided informed consent before participation. The confidentiality of participants’ personal information was maintained, and all data were anonymized to protect privacy. Additionally, the data were stored electronically on password-protected computers in a secure office. Access was restricted to the principal investigator (PI) to ensure confidentiality and data integrity.

### 2.6. Statistical Analysis

Statistical analysis was conducted using SPSS software, version 28 (IBM Corp., Armonk, NY, USA). Descriptive statistics were used to summarize the data. Continuous data are reported as means and standard deviations, while categorical data are presented as frequencies and percentages. Chi-square tests were applied to categorical data to examine associations between different factors. Fisher’s exact test was used when expected cell counts were less than five to ensure the validity of the results. For continuous data, independent *t*-tests or analysis of variance (ANOVA) were used, depending on the number of groups compared. All statistical tests were performed with a significance level of 0.05, and results were reported with 95% confidence intervals.

## 3. Results

The demographic characteristics of the recruited Umrah pilgrims are presented in Table 1. The study recruited 1138 participants. The mean age of the participants was 34.7 ± 14.8 years. The majority of the participants were female (813, 71.4%). In terms of BMI, 47 (4.1%) were underweight, 447 (39.3%) had a normal BMI, 344 (30.2%) were overweight, and 296 (26.1%) were obese. The majority of participants were from the Middle East and North Africa (MENA) region (991, 87.1%), followed by South and Southeast Asia (52, 4.6%), Sub-Saharan Africa (51, 4.5%), and Europe and the Americas (44, 3.9%). Most participants lived in the MENA region (1041, 91.5%), with smaller numbers from Sub-Saharan Africa (19, 1.7%), South and Southeast Asia (21, 1.8%), and Europe (57, 5.0%). In terms of education level, 687 (60.4%) had university or higher education, 206 (18.1%) had secondary education, 121 (10.6%) had intermediate education, 68 (6.0%) had primary education, and 56 (4.9%) had no formal education. Regarding employment, 359 (31.5%) were employed, while 779 (68.5%) were not. Most had previously performed Hajj or Umrah (1112, 97.7%), while 26 (2.3%) had not.

The prevalence of chronic diseases among Umrah pilgrims is presented in Table 2. Endocrine disorders (102, 9.0%) and cardiovascular disease (91, 8.0%) were the most common chronic conditions. Other chronic diseases reported included lung disease (17, 1.5%), musculoskeletal disorders (7, 0.6%), chronic kidney disease (6, 0.5%), immunosuppressive illness (5, 0.4%), hematological disorders (4, 0.4%), irritable bowel syndrome (IBS) (4, 0.4%), liver disease (4, 0.4%), and cancer (2, 0.2%). Most participants reported no history of these chronic conditions, with percentages ranging from 91.0% to 99.8% for each condition.

The prevalence of foot diseases and the distribution of injuries among Umrah pilgrims are presented in Table 3. Foot diseases were reported by 524 participants (46.0%). Regarding the types of foot injuries, the most common were sprains or strains (213, 18.7%), followed by muscle pain and cramps (56, 4.9%), blisters (24, 2.1%), fractures (18, 1.6%), cuts or abrasions (14, 1.2%), and other injuries (72, 6.3%). Most participants reported no injuries for each of these categories, with percentages ranging from 81.3% to 98.8%. In terms of injury location, the most frequently affected areas were the leg (231, 20.3%), forefoot (203, 17.8%), arch (131, 11.5%), and heel (114, 10.0%). Less commonly affected areas included the toes (98, 8.6%), ankle (91, 8.0%), and knee (2, 0.2%), with the majority of participants not experiencing injuries in these areas (ranging from 79.7% to 99.8%).

The utilization of medical attention and preventive measures for foot injuries among Umrah pilgrims is presented in Table 4. Of the participants, only 55 (9.6%) sought medical attention for foot pain, while the majority, 520 (90.4%), did not. Regarding using preventive measures to avoid foot injuries during Umrah, 169 participants (14.9%) reported using such measures, whereas 969 (85.1%) did not. Among those using preventive measures, the most common types included using analgesics and/or ointments (69, 46.6%), wearing footwear (51, 34.5%), using a wheelchair (22, 14.9%), and avoiding crowds (6, 4.1%).

The association between the incidence of foot diseases and demographic characteristics among Umrah pilgrims is presented in Table 5. The prevalence of foot diseases did not show a statistically significant difference across nationalities (*p* = 0.06), with the highest prevalence among Sub-Saharan Africa (51.0%) and the lowest among South and Southeast Asia (34.6%). Gender also did not show a significant association with foot diseases (*p* = 0.47), with similar prevalence among males (44.3%) and females (46.7%). Age showed a trend toward a higher prevalence in senior pilgrims (54.1%), though this was not statistically significant (*p* = 0.07). BMI categories also did not show a significant association (*p* = 0.06), with similar prevalence across underweight (51.1%), normal (45.2%), overweight (45.1%), and obese (48.0%) individuals. Education level, however, was significantly associated with foot diseases (*p* = 0.03), with the highest prevalence among those with primary education (61.8%) and the lowest among those with intermediate education (35.3%). Employment status (*p* = 0.8) and previous performance of Hajj or Umrah (*p* = 0.1) did not significantly affect foot disease incidence.

The association between the incidence of foot diseases and comorbidities among Umrah pilgrims is presented in Table 6. There was no significant association between endocrine disorders and foot diseases (*p* = 0.8), with a similar prevalence among those with (47.1%) and without (45.9%) endocrine disorders. However, a significant association was found between chronic liver disease and foot diseases (*p* = 0.04), with all participants with chronic liver disease (100%) reporting foot diseases. Similarly, a significant association was observed between cardiovascular disease and foot diseases (*p* = 0.04), with a higher prevalence among those with cardiovascular disease (56.0%) compared to those without (45.2%). No significant associations were found for chronic kidney disease (*p* = 0.4), chronic lung disease (*p* = 0.2), immunosuppressive illness (*p* = 0.3), cancer (*p* = 0.2), musculoskeletal disorders (*p* = 0.4), or hematological disorders (*p* = 1.0) with the incidence of foot diseases.

## 4. Discussion

Foot health during pilgrimage is a significant concern, given the physical demands and vast numbers of pilgrims engaging in extensive walking and standing [23]. This study aimed to assess the prevalence of foot problems among Umrah pilgrims and investigate how these relate to demographic characteristics and comorbidities. The study was conducted throughout the 2024 G (1445 H) Umrah season at the Grand Mosque in Makkah, involving 1138 participants.

The findings highlight a substantial burden of foot problems among Umrah pilgrims, with sprains and strains emerging as the most prevalent issues. Despite this, medical care was rarely sought, and preventive measures were largely neglected. Among those who took precautions, using analgesics and ointments was the most common approach. Additionally, foot problems were more frequently observed among individuals with lower education levels and those with chronic liver or cardiovascular conditions, underscoring the influence of demographic and health-related factors on foot health during pilgrimage.

The prevalence of chronic diseases in this study was relatively low, with endocrine disorders (9.0%) and cardiovascular diseases (8.0%) being the most commonly reported. This result is lower than findings from other studies conducted on Hajj pilgrims, where chronic conditions such as hypertension and diabetes were more prevalent [24]. These comparisons are contextual; Hajj and Umrah differ in timing, participant demographics, and health profiles. For example, a study by Mahomed et al. (2024) reported that 47% of the pilgrims had a chronic disease, with hypertension (39%) being the most common chronic disease, followed by diabetes (14%) [25]; in addition, Alsofayan’s study demonstrated diabetes prevalence at 49% and hypertension at 40%, both of which are major contributors to foot complications [26]. The discrepancy between these studies may arise from the age differences in the sample populations. Hajj pilgrims tend to be older (Mahomed et al., 2024) [25], and older individuals generally have a higher prevalence of chronic diseases like hypertension and diabetes, which are significant risk factors for foot complications (e.g., diabetic foot ulcers). Additionally, Umrah pilgrims typically undertake the pilgrimage at a younger age or may be in generally better health than those performing Hajj, explaining our study’s lower reported prevalence of chronic diseases.

The 46.0% prevalence of foot diseases found in this study is consistent with Sridhar et al. (2015), who reported that 31% of Hajj pilgrims experienced foot ailments [27]. Moreover, Alshehri et al. (2021) reported that musculoskeletal pain was most commonly reported in the ankle/foot (38.34%) and leg (29.89%) [28]. This is also similar to the findings of Almqaiti et al. (2024), who studied the prevalent foot injuries among pilgrims during the 2023 Hajj season and reported that blisters accounted for most injuries (61.5%), followed by ulcers (20.9%), indicating that physical exertion during pilgrimage contributes significantly to foot injuries [12]. The high prevalence of foot problems in this study and the other studies (Sridhar et al. (2015), Alshehri et al. (2021), and Almqaiti et al. (2024) [12,27,28] can be attributed to the intense physical activities involved in Umrah, including long hours of walking and standing, which put considerable strain on the feet. The similarity in results between these studies reflects everyday biomechanical stressors faced by pilgrims regardless of geographical location or specific pilgrimage type. However, the lower incidence of blisters in our study (2.1%) compared to Almqaiti et al. (2024) [12] could be due to differences in weather conditions, footwear choices, or foot hygiene practices. Pilgrims in our study may have used more suitable footwear or engaged in preventive measures, reducing the likelihood of blister formation [12].

One of the most striking findings of this study was that only 9.6% of participants sought medical attention for foot pain, despite the high prevalence of foot problems. Similarly, 85.1% did not use preventive measures, highlighting a significant gap in foot health awareness and self-care practices. These findings are consistent with previous research on Umrah pilgrims. A study by Tobaiqy et al. (2021) [29] assessing the practice of preventative measures among Umrah pilgrims in Saudi Arabia, 1440H-2019, found that although the experiences of the preventative measures among pilgrims in terms of health education, vaccinations, and hygienic practices were at times positive, their study identified several issues. These included the following preventative measures: immunizations, particularly meningitis and poliomyelitis vaccines, and using face masks in crowded areas [29]. Furthermore, Quadri et al. (2020) highlighted a similar trend in their study, where hajj pilgrims were found to show poor compliance with preventive protocols [30]. Similarly, Alqahtani et al. (2016) [31] reported that, despite high vaccination rates and widespread hand hygiene practices among Australian Hajj pilgrims, the overall uptake of preventive measures remained inconsistent. Consequently, the level of preventive care was suboptimal, suggesting room for improvement in educating and motivating pilgrims to fully adopt these measures [31]. Regarding seeking medical health, Sindy et al. (2015), assessing the pattern of patients and illnesses encountered at one health facility at Arafat on the second day of Hajj, reported that only those needing acute intervention seek medical advice during transit, and the general tendency was not to seek medical advice for problems that could be deferred while in transition, despite the presence of advanced health care facilities in Arafat, even for a day [32]. Cultural and religious factors may influence the reluctance to seek medical care, where pilgrims prioritize completing their religious duties over addressing health concerns. Additionally, lack of awareness regarding available healthcare services may contribute to the low healthcare-seeking behavior. Although the Ministry of Health of the Kingdom of Saudi Arabia has published information about Pilgrim’s Health on its website, including advice on falls and ankle sprains during Hajj [33], many pilgrims still do not take these preventive measures. The low adoption of preventive measures may stem from insufficient health education, as many pilgrims are unaware of the importance of proper footwear, foot hygiene, and early symptom management. Addressing these issues through targeted health campaigns and improved access to medical services during pilgrimage could enhance foot health and reduce the burden of foot-related conditions among pilgrims.

This study found that lower education levels were significantly associated with foot diseases (*p* = 0.03). This finding is consistent with studies such as that conducted by Alrashed et al. (2024) [34], who evaluated diabetic foot care knowledge and practices at the education level. Alrashed et al. reported that patients with lower education levels exhibited more diabetic foot complications than patients with higher education levels [34]. Similarly, Stolt et al. (2022) reported that lower educational attainment was associated with poorer foot health [35]. A possible explanation for the association between lower educational levels and foot diseases could be that individuals with lower education may have limited awareness of proper foot care practices, the importance of early symptom recognition, and the need for preventive measures. Lower health literacy may also contribute to reduced adherence to foot hygiene, appropriate footwear selection, and timely medical consultations. Additionally, individuals with lower education levels may have socioeconomic constraints that limit access to quality footwear and healthcare services, further increasing their risk of developing foot diseases.

This study found a significant association between chronic liver disease (*p* = 0.04) and cardiovascular disease (*p* = 0.04) with foot diseases. A connection between a diabetic foot infection and cardiovascular disease has been discovered through several investigations (Bertoluci et al. (2017), Shariful Islam et al. (2021), and Domingueti et al. (2016)) [36,37,38]. Similarly, A systematic review and meta-analysis by Chin et al. (2024) reported that diabetic foot ulcers are associated with major adverse cardiac events [39]. Moreover, X et al. (2023) reported that liver disease was associated with an increased risk of peripheral neuropathy [40]. The association between chronic liver disease and foot diseases may be less commonly discussed but could be due to fluid retention and poor circulation, which can exacerbate foot conditions. The association between cardiovascular disease and foot ailments is well-established, as reduced blood circulation impairs foot healing and increases the risk of ulceration [41]. Any differences in the presence of comorbidities in our study compared to other studies may be due to sample variations—for instance, age and health profiles of the participants could play a role in these findings.

## 5. Strengths and Limitations

This study can be evaluated in light of its strengths and limitations. The strengths of this study include its large sample size of 1138 participants, significantly exceeding the original target of 385, which enhances the generalizability of the results and provides a robust dataset for assessing the prevalence of foot problems among Umrah pilgrims. A comprehensive data collection approach was employed through a well-structured questionnaire that captured relevant information, including demographic characteristics, chronic diseases, foot injuries, and preventive measures, facilitating a multifaceted assessment of foot health during the pilgrimage. Including a diverse pilgrim population from various nationalities, educational backgrounds, and health statuses enhances the representativeness of the findings, allowing for a broader understanding of the prevalence and factors associated with foot health among different groups of pilgrims. The study also adhered to ethical guidelines, ensuring informed consent, voluntary participation, and confidentiality of participants’ responses, which enhances its credibility and respects participants’ rights. Finally, the findings hold significant practical relevance for improving foot health during Umrah, providing valuable insights into the prevalence of foot-related health issues and identifying gaps in preventive care that can inform future interventions and awareness campaigns for pilgrims. However, the study has several limitations that should be acknowledged. Firstly, the cross-sectional design provides only a snapshot of foot health during the pilgrimage and does not establish causality; longitudinal studies would be necessary to assess the long-term impact of pilgrimage on foot health and determine causal relationships between demographic factors, comorbidities, and foot diseases. While research assistants provided standardized guidance during data collection to improve accuracy, the study ultimately relied on assisted self-reported data, which may still lead to underreporting or misclassification of conditions. Future studies could strengthen findings with clinical examinations or medical record verification. Additionally, while the questionnaire was carefully translated by bilingual researchers, formal validation procedures such as back-translation and pilot testing were not conducted, which should be addressed in future research. Furthermore, while the study included pilgrims aged 18 years and older to reflect the adult pilgrim population, variations in healing capacity and foot injury susceptibility across age groups (e.g., younger vs. elderly pilgrims) were not specifically analyzed. This warrants further investigation in future age-stratified studies. The exclusion of pilgrims with severe pre-existing foot conditions, such as diabetic foot ulcers or active infections, may limit the applicability of the findings to the broader pilgrim population, particularly those with comorbidities that exacerbate foot problems. Lastly, recall bias is a concern, as participants may not accurately remember the frequency and severity of foot conditions, especially if they did not seek immediate medical attention.

### Clinical and Practical Implications

Understanding the burden of foot problems among Umrah pilgrims has several important implications. Firstly, it can aid in developing targeted healthcare interventions and preventive strategies, such as education campaigns on appropriate footwear, better medical screening for high-risk individuals, and improved healthcare access within pilgrimage sites. Secondly, it contributes to the broader field of mass gathering medicine by offering insights into health risks associated with prolonged physical activity in high-density environments. Ultimately, the findings of this study aim to support policymakers, healthcare providers, and religious authorities in ensuring a safer pilgrimage experience for millions of Muslims worldwide.

## 6. Conclusions

This study confirms that foot injuries are a pervasive yet under-addressed issue among Umrah pilgrims, affecting 46% of participants, predominantly as sprains, muscle pain, and forefoot damage. The association between foot problems and lower education levels underscores socioeconomic disparities in foot health awareness, while links to chronic diseases highlight the vulnerability of medically complex pilgrims. Notably, <10% sought medical care despite injury prevalence, which may reflect pilgrims’ prioritization of ritual completion over self-care or unrecognized severity of early symptoms. These findings demonstrate that foot health during Umrah is influenced by education levels, chronic disease status, and care-seeking behaviors.

### Future Recommendations

In light of the study’s findings, several actionable recommendations can be made to address the prevalent foot problems among Umrah pilgrims. The government and health authorities could implement robust foot care programs, including dedicated clinics at the Grand Mosque to provide immediate medical attention and preventive care such as foot assessments, footwear advice, and treatment for common injuries. Increasing public awareness through digital platforms, religious institutions, and travel agencies is also essential for educating pilgrims on proper footwear choices, self-care practices, and the importance of seeking medical attention when needed. Encouraging the use of appropriate footwear, foot hygiene, and essential foot care supplies could further reduce the burden of foot-related health issues. Additionally, collaboration with manufacturers and retailers to promote affordable, suitable footwear as part of pilgrimage packages may help pilgrims better prepare for the physical demands of Umrah. From a research perspective, further studies are needed to explore the underlying causes of foot problems, including footwear, walking patterns, and environmental influences within the Grand Mosque. Investigating barriers that prevent pilgrims from seeking medical attention or adopting preventive measures—such as cultural, socioeconomic, or awareness-related factors—could provide valuable insights. A mixed-methods approach combining quantitative data with qualitative interviews may offer a deeper understanding of pilgrims’ experiences and challenges in managing foot health. Moreover, evaluating the effectiveness of interventions, such as educational campaigns and foot care clinics at pilgrimage sites, could help bridge knowledge gaps and reduce foot-related health problems among pilgrims.

## Figures and Tables

**Table 1:** Demographic characteristics of recruited Umrah pilgrims (*n* = 1138).

Demographic Data	*n* (%)
Age	34.7 ± 14.8
**Gender**	
Male	325 (28.6)
Female	813 (71.4)
**BMI**	
Underweight	47 (4.1)
Normal	447 (39.3)
Overweight	344 (30.2)
Obese	296 (26.1)
**Nationality**	
Middle East and North Africa (MENA)	991 (87.1)
Sub-Saharan Africa	51 (4.5)
South and Southeast Asia	52 (4.6)
Europe and Americas	44 (3.9)
**Country residence**	
Middle East and North Africa (MENA)	1041 (91.5)
Sub-Saharan Africa	19 (1.7)
South and Southeast Asia	21 (1.8)
Europe	57 (5.0)
**Education level**	
No formal education	56 (4.9)
Primary education	68 (6.0)
Intermediate education	121 (10.6)
Secondary education	206 (18.1)
University/Higher education	687 (60.4)
**Employment**	
Yes	359 (31.5)
No	779 (68.5)
**Previously performed Hajj or Umrah**	
Yes	1112 (97.7)
No	26 (2.3)

**Table 2:** Prevalence of chronic diseases among Umrah pilgrims (*n* = 1138).

Chronic Disease	Yes	No
**Endocrine Disorders**	102 (9.0)	1036 (91.0)
**Cardiovascular disease**	91 (8.0)	1047 (92.0)
**lung disease**	17 (1.5)	1121(98.5)
**Musculoskeletal Disorders**	7 (0.6)	1131(99.4)
**Chronic kidney disease**	6 (0.5)	1132(99.5)
**Immunosuppressive illness**	5 (0.4)	1133 (99.6)
**Hematological Disorders**	4 (0.4)	1134 (99.6)
**IBS**	4 (0.4)	1134 (99.6)
**Liver disease**	4 (0.4)	1134 (99.6)
**Cancer**	2 (0.2)	1136 (99.8)

**Table 3:** Prevalence of foot diseases and distribution of injuries among Umrah pilgrims (*n* = 1138).

Variable	Yes	No
Foot diseases	524 (46.0)	614 (54.0)
**Type of foot injury experienced**		
Sprains or strains	213 (18.7)	925 (81.3)
Muscle pain and cramps	56 (4.9)	1081 (95.1)
Blisters	24 (2.1)	1114 (97.9)
Fractures	18(1.6)	1120 (98.4)
Cuts or abrasions	14 (1.2)	1124 (98.8)
Other	72 (6.3)	1066 (93.7)
**Foot injury location**		
Leg	231 (20.3)	907 (79.7)
Forefoot	203 (17.8)	935 (82.2)
Arch	131 (11.5)	1007 (88.5)
Heel	114 (10.0)	1024 (90.0)
Toes	98 (8.6)	1040 (91.4)
Ankle	91 (8.0)	1047 (92.0)
Knee	2 (0.2)	1136 (99.8)

**Table 4:** Utilization of medical attention and preventive measures for foot injuries among Umrah pilgrims (*n* = 1138).

Variable	Frequency	Percent
**Sought medical attention for foot pain**		
Yes	55	9.6
No	520	90.4
**Using preventive measures to prevent foot injuries during Umrah**		
Yes	169	14.9
No	969	85.1
**Type of preventive measure used**		
Wheelchair	22	14.9
Footwear	51	34.5
Using analgesics and/or ointments	69	46.6
Avoiding crowd	6	4.1

**Table 5:** Association between incidence of foot diseases and demographic characteristics among Umrah pilgrims.

Variable		Foot Diseases		*p* Value
		Yes	No	
**Nationality**	**Middle East and North Africa (MENA)**	466 (47.0)	525 (53.0)	0.06
	**Sub-Saharan Africa**	26 (51.0)	25 (49.0)	
	**South and Southeast Asia**	18 (34.6)	34 (65.4)	
	**Europe and Americas**	14 (31.8)	30 (68.2)	
**Gender**	**Male**	144 (44.3)	181 (55.7)	0.47
	**Female**	380 (46.7)	433 (53.3)	
**Age**	**Youth/Young Adult**	170 (49.0)	177 (51.0)	0.07
	**Early Adulthood**	117 (41.8)	163 (58.2)	
	**Midlife**	89 (40.8)	129 (59.2)	
	**Late Adulthood:**	128 (50.2)	127 (49.8)	
	**Senior**	20 (54.1)	17 (45.9)	
**BMI**	**Underweight**	24 (51.1)	23 (48.9)	0.06
	**Normal**	202 (45.2)	245 (54.8)	
	**Overweight**	155 (45.1)	189 (54.9)	
	**Obese**	142 (48.0)	154 (52.0)	
**Education level**	**No formal education**	30 (53.6)	26 (46.4)	0.03 *
	**Primary education**	42 (61.8)	26 (38.2)	
	**Intermediate education**	43 (35.3)	78 (64.5)	
	**Secondary education**	104 (50.5)	102 (49.5)	
	**University/Higher education**	305 (44.4)	382 (55.6)	
**Employment**	**Yes**	164 (45.7)	195 (54.3)	0.8
	**No**	360 (46.2)	419 (54.8)	
**previously performed Hajj or Umrah**	**Yes**	516 (46.4)	596 (53.6)	0.1
	**No**	8 (30.8)	18 (969.2)	

* Statistical significance is indicated by *p*-values less than 0.05.

**Table 6:** Association between incidence of foot diseases and comorbidities among Umrah pilgrims.

Variable		Foot Diseases		*p* Value
		Yes	No	
**Endocrine Disorders**	**Yes**	48 (47.1)	54 (52.9)	0.8
	**No**	476 (45.9)	560 (54.1)	
**Chronic liver disease**	**Yes**	4 (100)	0 (0.0)	0.04 * (F)
	**No**	520 (45.9)	614 (54.1)	
**Chronic kidney disease**	**Yes**	4 (66.7)	2 (33.3)	0.4
	**No**	520 (45.9)	612 (54.1)	
**Chronic lung disease**	**Yes**	10 (58.8)	7 (41.2)	0.2
	**No**	514 (45.9)	607 (54.1)	
**Immunosuppressive illness**	**Yes**	6 (66.7)	3 (33.3)	0.3 (F)
	**No**	518 (45.9)	611 (54.1)	
**Cancer**	**Yes**	2 (100)	0 (0.0)	0.2 (F)
	**No**	522 (46.0)	614 (54.0)	
**Cardiovascular disease**	**Yes**	51 (56.0)	40 (44.0)	0.04 *
	**No**	473 (45.2)	574 (54.8)	
**Musculoskeletal Disorders**	**Yes**	2 (28.6)	5 (71.4)	0.4 (F)
	**No**	522 (46.2)	609 (53.8)	
**Hematological Disorders**	**Yes**	2 (50.0)	2 (50.0)	1.0 (F)
	**No**	522 (46.0)	612 (54.0)	
	**No**	423 (43.7)	546 (6.3)	

* Statistical significance is indicated by *p*-values less than 0.05. F: Fisher’s exact test was used for variables with small sample sizes or sparse data to determine *p*-values.

## Data Availability

The dataset used and/or analyzed during the current study is available from the corresponding author on reasonable request.
